# Ear-body lift and a novel thrust generating mechanism revealed by the complex wake of brown long-eared bats (Plecotus auritus)

**DOI:** 10.1038/srep24886

**Published:** 2016-04-27

**Authors:** L. Christoffer Johansson, Jonas Håkansson, Lasse Jakobsen, Anders Hedenström

**Affiliations:** 1 Dept. Biology, Lund University, Ecology building, SE-223 62 Lund, Sweden; 2 Dept. Biology, University of Southern Denmark, Denmark

## Abstract

Large ears enhance perception of echolocation and prey generated sounds in bats. However, external ears likely impair aerodynamic performance of bats compared to birds. But large ears may generate lift on their own, mitigating the negative effects. We studied flying brown long-eared bats, using high resolution, time resolved particle image velocimetry, to determine the aerodynamics of flying with large ears. We show that the ears and body generate lift at medium to cruising speeds (3–5 m/s), but at the cost of an interaction with the wing root vortices, likely reducing inner wing performance. We also propose that the bats use a novel wing pitch mechanism at the end of the upstroke generating thrust at low speeds, which should provide effective pitch and yaw control. In addition, the wing tip vortices show a distinct spiraling pattern. The tip vortex of the previous wingbeat remains into the next wingbeat and rotates together with a newly formed tip vortex. Several smaller vortices, related to changes in circulation around the wing also spiral the tip vortex. Our results thus show a new level of complexity in bat wakes and suggest large eared bats are less aerodynamically limited than previous wake studies have suggested.

Bats represent more than 20% of all mammalian species, span a size range from 2 to 1600 g and, with few exceptions, occur globally^1^. They are the only mammals capable of powered flight and one of only two groups (Odontocetes being the other) to evolve echolocation, i.e. the ability to localize objects by listening to the returning echoes of emitted sound pulses^2^. In all likelihood, it is the interplay between powered flight and echolocation that account for the evolutionary success of bats. Together, flight and echolocation, have enabled bats to forage in the night sky, a niche that presumably was relatively unexploited until bats emerged about 65 My ago. At the same time, the ability to fly and echolocate efficiently is not without conflict. While large ears are beneficial for echolocation and prey detection they most likely affect the aerodynamic performance negatively^3^,^4^.

Bats display a large variation in airframe “design”^5^. Their morphology is closely related to the habitat and ecological niche they occupy. For example, bats with long slender wings (efficient for transportation flight) are primarily found in open spaces, while species having large broad wings (favoring high maneuverability) tend to inhabit more cluttered habitats^5^. The emitted echolocation calls, as well as ear morphology, are likewise related to ecology^6^, reflecting the complexity of the acoustic scene and the perceptual task. Fast flying open-space hawking bats tend to emit loud, long duration calls and have relatively small ears, while slow flying gleaning bats emit short quiet calls and have relatively large ears^7^,^8^,^9^. There is thus a correlation between large ears and slow flight, suggesting ears may limit flight speed.

To determine the aerodynamics of a flying animal we study its wake, which is the aerodynamic footprint, heralding information about the forces generated by the animal. Compared to birds, bats generate relatively strong wing root vortices, indicative of reduced lift generation across the body region^4^,^10^,^11^,^12^,^13^,^14^,^15^,^16^,^17^. This is likely a consequence of the ears disrupting the lift generating flow, which reduces the downwash behind the body, compromises span efficiency, and as a consequence increases induced drag^3^,^4^. All bat species studied to date have relatively small ears and occupy a very limited portion of the bat morphospace^18^. Large eared bats may be more aerodynamically restricted (i.e. unable to fly as fast) than small eared bats, but studies of model bat bodies find that large ears may mitigate the negative effect of ears by using the ears to generate lift^19^,^20^. The consequences of flying with large ears thus calls for a detailed aerodynamic study of freely flying large-eared bats.

Bats differ from birds and insects regarding the wing morphology and kinematics^18^. The compliant skin membrane stretched by articulated fingers allow a higher control of the wing shape and motions than found in the other groups of flying animals^21^. Likewise, several studies suggest that bat wakes are more complex than those of birds^4^,^12^, exhibiting unique characteristics (for recent reviews see^18^,^22^,^23^). For example, bats can actively control the wing twist, enabling the inner and outer wing to operate with opposite circulation simultaneously^24^. The wing morphology also allows for a dynamic control of the forces^25^ and provides a high diversity of flight control mechanisms^21^. As an example, a strong pitching motion has been described to occur at the transition between the upstroke and downstroke in slow flying brown long-eared bat^26^. This wing rotation has the potential to add to the circulation (hence lift) of wings^27^,^28^, and contribute useful weight support at slow flight speeds. By studying the details of the wake, we can test how this and other flight mechanisms function aerodynamically.

To unravel the highly complex bat wakes, time resolved measurements of high resolution are required. Although, previous wake studies have revolutionized our understanding of bat flight^12^,^29^, many questions regarding for example the dynamics of the transitions between upstrokes and downstrokes have not been fully resolved. Unlike birds, the outer wing of bats generate reversed vortex loops towards the end of the upstroke at cruising speed, indicative of thrust generation at the cost of negative weight support^4^,^10^,^11^,^12^,^13^,^14^,^15^,^16^,^17^. The inner wing retains normal circulation during the upstroke and how the transition to the downstroke function in this complex situation is not clear. Here we study brown long-eard bats (*Plecotus auritus*), using state of the art particle image velocimetry, of higher temporal and spatial resolution than any previous bat wake study, to determine the effect of large ears and novel characteristics of aerodynamics in bat flight.

## Results

### Lift generation

We trained three wild-caught *P. auritus*, to fly at a feeder in the wind tunnel at Lund University, Sweden. The bats were flown at 1–5 m/s and we measured the flow in the wake closely behind the bats using time resolved stereo Particle Image Velocimetry (PIV). We used average weight support for each sequence as a control to determine if we had captured the relevant vortices in the wake. On average, weight support was 99 ± 9.2% (mean ± SD, N = 26) in the sequences used (Fig. S3a). The momentary weight support measured in the wake change throughout the wingbeat differently at different speeds. At U = 1 and 2 m/s the wakes of successive wingbeats overlap and weight support measured from the wake was never below zero during a wingbeat period (Fig. S3b,c). However, at 3–5 m/s weight support was below zero during parts of the upstroke (Fig. S3d–f).

### Wake topology

**Figure 1:**
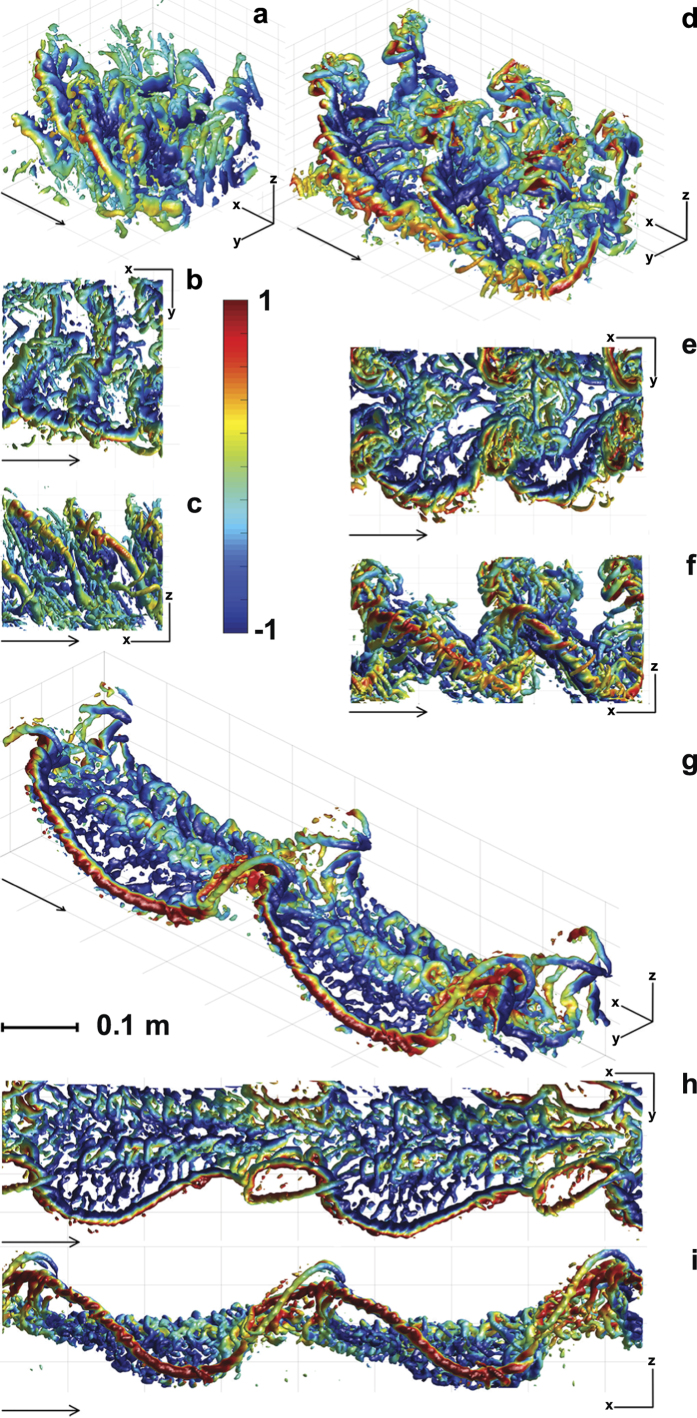
Iso-surface plots of the Q-criterion (2500) colored by vertical flow speed (red upwards, blue downwards), at speeds U = 1 m/s (a–c), U = 2 m/s (d–f) and U = 4 m/s (g–i) seen obliquely from above and in front (a,d,g), from above (b,e,h) and from the side (c,f,i). Arrows represent flight direction and all panels are scaled according to the scale bar in panel (**g**). The color is scaled relative to mean absolute vertical speed + 3*SD of the vertical speed. Maximum red color thus represents 2.8 m/s at U = 1 m/s, 1.3 m/s at U = 2 m/s and 1.1 m/s at U = 4 m/s.

The wake topology varied across flight speeds. At the lowest speed, 1 m/s, we find a strong tip vortex associated with the downstroke (Fig. 1a–c). The tip vortex is connected with a start vortex, a spanwise vortex shed shed at the beginning of the downstroke as the wing starts generating lift, which connects above the body to the start vortex of the other wing (Fig. 1a–c). The tip vortex shows a spiraling structure, with smaller structures added to the tip vortex as the downstroke progresses. The wings are moved far forward relative to the body at the end of the downstroke, although we do not observe any distinct stop vortex. At this speed the wing is flipped up-side down during the upstroke and reverses direction relative to the air. During the upstroke, wing span is reduced by flexing the elbow, which results in the wake of the upstroke being formed “inside” the wake of the downstrokes. As a result, the upstroke wake is squeezed between consecutive downstroke wakes. It is however possible to discern relatively weak tip and a root vortices, with a steeper angle relative to the horizon than the wake of the downstroke (Fig. 1a–c).

**Figure 2:**
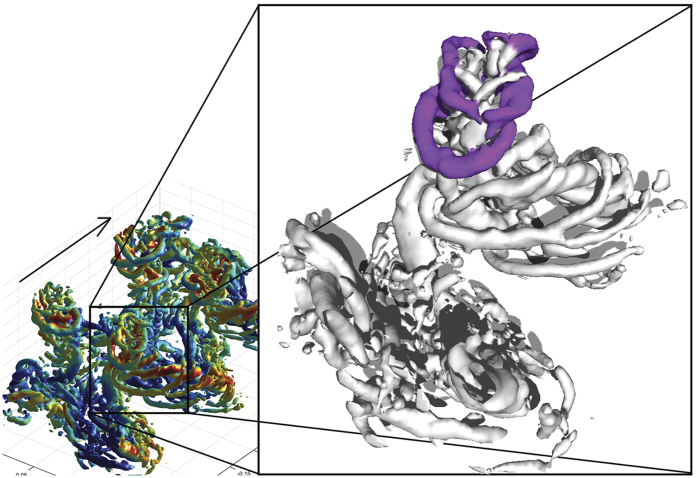
Iso-surface plot of Q-criterion (2500) showing the vortices generated at the transition between upstroke and downstroke at 2 m/s, viewed obliquely from above and behind. Vortex structures showing upwards and backwards induced flow, formed as the wing performs a pronating, pitch down, motion at the transition are colored purple. Flight direction is indicated by arrow. Rotatable 3D image is available in the SI.

At 2 m/s the inner part of the wing moves forward relative to the air during the upstroke, while the outer part of the wing moves upwards and slightly backwards. In addition, during the end of the downstroke the wing is moved far forwards relative to the body. There is a distinct start vortex, connected to a root vortex, formed at the start of the downstroke (Fig. 1d–f). We also find a vortex of opposite spin to the tipvortex formed somewhere near the mid wing, which is connected to a spanwise start vortex and a distal vortex forming one of the entwined structures of a spiraling tip vortex (Figs 1d–f and S5). During the downstroke additional spanwise vortices connect to the spiraling tip vortex. The wake of the outer part of the wing forms a very complex wake during the upstroke, where the tip vortex of the downstroke becomes reduced in strength towards the end of the downstroke, but does not disappear completely and instead sheds at a position approximately mid wing. This mid wing vortex is oriented almost straight up during the upstroke after which it forms one of the cores of the spiraling tip vortex of the next downstroke (Fig. 1d–f). Towards the end of the downstroke, vortices, reflecting reduced circulation, are shed at the outer wing. These vortices spiral around the “tip vortex” of the wing. As the upstroke progresses the outer wing first moves upwards and then backwards, and at the time of transition to the downstroke, the wing is pronated. The wake found during the first part of the upstroke is highly variable. The outer wing starts the upstroke in a forward position relative to the body, resulting in the upstroke wake being “run over” by the wake of the inner wing e.g. the vortices related to the beginning of the upstroke are deformed by the root vortex of the proceeding downstroke. At the transition of the upstroke/downstroke we find a reversed vortex structure of one to three vortex loops (reflecting negative weight support and thrust generation) positioned above the start vortex of the downstroke (Figs 2 and S1). This structure is found in all of the seven sequences analyzed. However, although these are the main structures repeatedly occurring in the wake the complexity and variation between wingbeats make interpretation a challenge.

**Figure 3:**
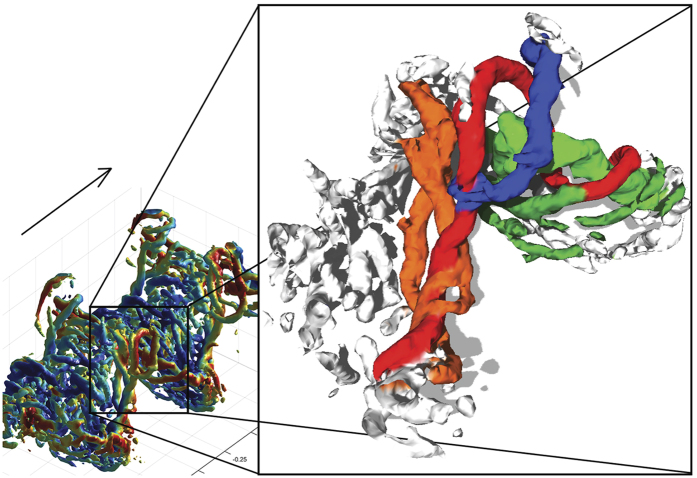
Iso-surface plot of Q-criterion (2500) showing the details of vortices generated at the transition between upstroke and downstroke at 3 m/s, viewed obliquely from above and behind. The tip vortex (red), reduces in strength during the upstroke resulting in shedding of stop vortices (orange). Towards the end of the upstroke the wing tip sheds a tip vortex (blue) of opposite sense of rotation to the normal tip vortex. At the beginning of the next downstroke, a start vortex is shed (light green). The circulation builds up during the downstroke as indicated by additional start sense vortices (dark green) being shed. The resulting tip vortex constitutes several vortices spiraling around each other. Flight direction indicated by arrow. Rotatable 3D image is available in the SI.

**Figure 4:**
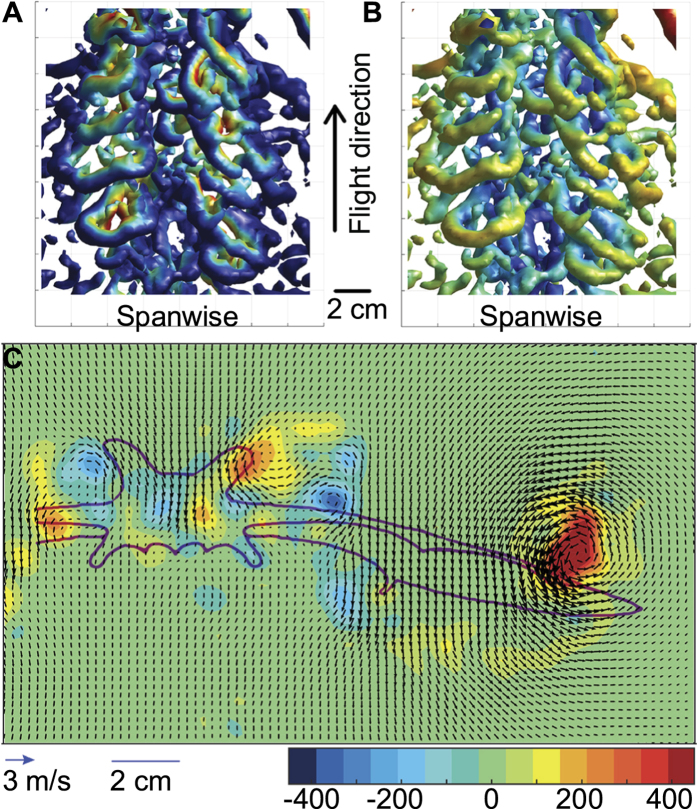
Iso-surface plot of Q-criterion (2000) showing the body/wing root wake at 4 m/s during a fraction of the downstroke seen from above, colored by vertical speed (red upward, blue downward) (A) and streamwise flow relative to free stream (red backward, blue forward) (B). Multiple vortex ring structures form during a wing beat showing upward and forward induced flow, indicating negative weight support and drag. Flight direction is indicated by the arrow between A and B. Vector field, smoothed once, of the induced flow at mid downstroke of a bat flying at 4 m/s (**C**). Colors represent streamwise vorticity, with red being counter clockwise and blue clockwise rotation. Note the clear downwash behind the body and the upwash between the body and the wing root vortices.

At medium to cruising speeds (3 m/s and above) the wakes are topologically similar (Fig. 1g–i). At these higher speeds, a tip vortex is trailing the wing tip motion during the downstroke (Fig. 1g–i). The tip vortex shows a spiraling appearance with smaller vortices entwining the main tip vortex, which connects to smaller spanwise ‘start’ vortices during the downstroke. During the upstroke the tip vortex moves proximally, which is accompanied by the shedding of stop vortices. At approximately mid upstroke a vortex of reversed sign relative to the tip vortex forms at the wing tip (Fig. 1g–i). The previous tip vortex is maintained approximately half way along the wing. Together these vortices encircle a volume of upwash, forming a reversed vortex loop (Figs 3, S2 and S6). At the center of the body we find an area of relatively high downwash (Fig. 4). During the downstroke we find a vortex structure, showing reduced streamwise flow and upwash, between the tip vortex and the body wake (Figs 1g–i and 4). This structure shows a cyclic pattern, resulting in what appears to be discrete loops, about 5–7 structures generated during each downstroke and is visible in all the 17 sequences analyzed for 3 m/s and above.

## Discussion

Contrary to all previous studies on bats, we find that the body region of brown long-eared bats generates substantial downwash. This shows that the ears do not disrupt the flow over the body of *P. auritus*, as in other bat species, and supports the view that large ears provide lift that contributes to weight support. However, the relevance of the tail membrane for the force generation of the body needs further investigation. Furthermore, our unprecedented recording resolution revealed multiple novel wake structures, i.e. spiraling tip vortices, ear-wing root interaction vortices, and wing pitch vortices. Despite the novel features of the wake, the overall wake structure is consistent with that found in previous studies^10^,^11^,^12^,^13^,^17^,^30^, and the characteristic features attributed to bat wakes are also found here; distinct wing root vortices and end of upstroke reversed vortex loops at cruising speed^12^. Also at the lowest speed, the wake of *P. auritus* resembles the hovering wake of other bat species^31^. However, our results show that the wake of bats is even more complex than previously recognized and calls for revisiting previously studied species, as well as other species, to determine how general the novel wake structures found here are.

Ears are likely to contribute to the relatively low lift to drag ratio found in bats compared to birds, by reducing the lift generated by the body, increasing the parasite drag and reducing the span efficiency^3^,^4^. As a consequence we expected the large ears of *P. auritus* to generate substantial drag and little lift, especially at cruising speed. Instead we found a distinct downwash behind the body (Fig. 4), which is contrary to other small-eared bat species^10^,^11^,^17^,^30^. This shows that the ears/body/tail generates significant lift, consistent with results from bat models with big ears (e.g.^19^,^20^). However, since *P. auritus* also possess a relatively large tail membrane, separating out the effect of ears and tail is not possible, although model studies finds a relatively small effect of the tail membrane^32^. During the wingbeat we occasionally found two vortex pairs, stacked on top of each other in the body region (Fig. 4). A likely explanation would be one set of vortices generated by the ears and one set by the body/tail, but clarifying this requires measurements of the flow directly behind the ears or physical or computational model studies.

The ears point forwards and slightly laterally suggesting the aerodynamics to be similar to that in forward swept wings or reversed delta wings. These wings induce an inward flow, contracting the wake i.e. moving the tip vortices closer together than in normally swept wings^33^. The wake generated by the ears will interact with the flow over the wings, and to produce a relatively narrow wake from the ears is beneficial since it reduces the area of the wings affected by the ear wake. The wing root vortices are positioned relatively far out on the arm-wing, in contrast to other species where they occur close to the wing root (Fig. 4)^10^,^11^,^17^,^30^. This indicates that the innermost part of the wing generates less lift than would otherwise be the case. Between the ear-tip/body vortex and the wing-root vortex we find a pulsed induced upwash and forward flow reflecting negative weight support and drag during the downstroke at cruising speed (Fig. 4). We suggest these structures are, at least partly, a result of an interaction between tip vortices from the ears and root vortices from the wings. However, the upward flow forms discrete vortex rings, signaling unsteady behavior. When visually inspecting videos of our bats flying at cruising speed we observe a fluttering trailing edge of the plagiopatagium, which may be responsible for, or a response to this unsteady flow. Regardless of the origin of these pulsed structures, they reflect an increased cost for the animals since they result in lower span/flap efficiency and potentially lower lift than would otherwise be possible.

The high temporal and spatial resolution of our PIV data allowed us to uncover details previously not noticed in the wakes of bats. One such example is the occurrence of spiraling tip vortices, which occur at all speeds (Fig. 1). When examining the details of the transition between the up- and down-stroke at 3 m/s, we note that these spiraling vortices form, partly, as a consequence of spanwise vortices connecting to the tip vortex (Fig. 3, green). During the wingbeat the wings are accelerating and the angle of attack of the wings increase during the beginning of the downstroke and the reverse is true during the transition to the upstroke^21^. Previous research has shown that the circulation of the tip vortices changes throughout the wingbeat^10^,^11^,^30^, which requires shedding of spanwise vorticity since the circulation of the wing/wake system must remain constant (Kelvin circulation theorem^34^). In the first bat wake studies using streamwise PIV planes^12^,^13^, this vorticity rolls up in discrete vortices with the same sign of rotation as the main start vortex during the beginning of the downstroke and opposite during the end of the downstroke and beginning of the upstroke. This observation is consistent with expectations from circulation changes related to wing acceleration and changes in angle of attack.

Spiraling tip vortices are also found in tethered desert locusts (*Shistocerca gregaria*)^35^, in computational modelling of flying insects (e.g.^36^) and heaving, rotating and accelerating 3D-plates^37^,^38^,^39^. In the presence of leading edge vortices, a spiraling tip vortex may be generated when a multi-cored leading edge vortex^40^ sheds and merges with the tip vortex^36^. Although our bats most likely use LEVs at low speeds^18^,^24^,^29^, this is unlikely the case at higher speeds^24^, and can therefore not explain our observed pattern. The suggested mechanism for the observed spiraling wing tip vortices in locusts is Kelvin-Heimholtz instability in the shear layer behind/over the insect wing. The instability results in the rollup of spanwise vortices at the trailing edge that are drawn into and orbits the tip vortex^35^. Since the Kelvin-Heimholtz instability could be responsible for the rollup of vortices also due to changes in circulation it is difficult to test if the same mechanism also applies to bats. A shear layer will always be present behind the wing, since the profile drag of the wing reduces the flow speed relative to the surrounding air. Kelvin-Heimholtz instability should therefore result in spanwise vortices regardless if the circulation around the wing changes or not. However, in the case of the moving plates^37^,^38^,^39^ the results point to the presence of spanwise vortices connecting to the tip vortex only when wings are accelerating, i.e. when circulation gradually increases. Consequently, we consider the spiraling tip vortex in *P. auritus* to be a result of a gradual change in circulation of the wing making it visible at the early stages of vortex rollup and as such would be expected also in other actively flying animals.

In addition to the smaller start and stop vortices connecting to the tip vortex, we find that one of the cores of the spiraling tip vortex connects to the tip vortex of the previous wingbeat (Figs 1, 2, 3). When we look in detail at the upstroke/downstroke transition (Fig. 3) we see that the tip vortex (Fig. 3, red) moves inwards along the span, and is maintained throughout the transition to form one of the cores of the tip vortex of the succeeding wing beat (this also applies at U = 2 m/s, Fig. 2). This pattern is clearly visible in 14 out of 17 cruising speed sequences and discernable in the remaining three sequences. This depicts a different origin of the spiraling tip vortex compared to that of desert locusts (and accelerating and heaving plates). In this case the spiraling tip vortex relates to the opposite sign of circulation of the inner and outer wing during the upstroke, which has so far not been described in other flying animals and is likely unique to bats. Whether these spiraling vortices have any significance for the animals or simply reflect the roll up of the tip vortices during gradually changing circulation around the wing remains open.

The wake at the lowest speed, 1 m/s, shows resemblance with the hovering wake of *Leptonycterus yerbabueanae*^31^. The downstroke dominates the wake and the upstroke is active, with the wing flipped up-side down, generating a relatively weak wake. However, in the wake it is clear that the start vortices of the two wings connect over the body of the bat and that the stop vortices (which should be in the streamwise direction due to the wings pointing almost straight forward at the end of the downstroke) are absent. In fact, in at least one sequence we see that the wing tip vortices of the left and right wings are connected at the end of the downstroke. This means that the bat generates a single vortex ring for the two wings. This wake structure deviates from the previously found wakes for slow flying bats^12^,^29^,^31^ and is likely the result of a merging of the stop vortices of each wing as the wings come close together at the end of the downstroke. This is unexpected though, considering the similarity in the wing motion between *L. yerbabueanae* and *P. auritus.*

In a narrow range of speeds, above hovering and below cruising, the conditions for generating aerodynamic forces with flapping wings provide specific challenges, e.g. upstroke provides little weight support^13^. The wings are flapped in a stroke plane inclined relative to the horizon, i.e. the wings move upwards and backwards relative to the body during the upstroke (e.g.^21^,^25^,^26^,^41^). In slow forward flight this results in the outer wing moving backwards relative to still air, while the inner wing is moving forwards. Hence, circulation can change sign along the span, as shown by simultaneous leading edge vortices of opposite circulation along the span^24^. However, the wing orientation suggests mainly horizontal forces during the upstroke. At 2 m/s we find the most complicated wake structure found in bat wakes so far^10^,^11^,^12^,^13^,^17^,^30^,^31^ (Figs 1d–f and 2). In addition to the complexity associated with the wing kinematics, the rather weak wake of the upstroke becomes deformed by the strong vortices of the downstroke. However, the measurements are made ~10 cm behind the bats, which gives a transition time between the bat and laser sheet of 0.05s at 2 m/s (63% of a wingbeat) and therefore we consider the deformations, other than wake convection, to be insignificant^35^. The wake at 2 m/s indicates a relatively low aerodynamic efficiency, due to a varying downwash distribution that departs from a uniform (ideal) downwash associated with maximum span efficiency. Although not quantitatively measured, it has been reported that bats struggle more when flying in this speed-range than either above or below^21^, supporting this view. However, it remains to be determined if these slow speeds result in a local maximum in the mechanical power for bat flight.

At the end of the upstroke a reversed vortex loop is generated (Fig. 2), similar in appearance to that found at higher speeds (Fig. 3). However, considering that the outer wing is flipped upside down during the upstroke at 2 m/s, this reversed vortex cannot have a similar origin as that at cruising speed, where it is caused by a negative angle of attack of the normally oriented wing moving upwards^21^,^25^,^26^. Norberg^26^ described a strong wing pronation (~90 degrees) at the end of the upstroke at 2.35 m/s in *P. auritus* (also present in our videos, but not quantified) and suggested the function to be akin to the wing flip described by Weis-Fogh^28^. As such, the pitch is expected to add to the circulation of the wing and contribute weight support. However, we found a negative weight support and thrust during this phase of the upstroke, showing a different process at work. We propose that the force is generated in a process akin to the c-start in fish. During a c-start the fish’s tail goes through a strong pitching motion that results in a high thrust force (e.g.^42^), which is reflected in the wake by a distinct vortex loop^43^,^44^. Similar wakes have also been observed in plates pitched through large angles (e.g.^45^,^46^) and models of the feet of swimming birds^47^. The wing pitch vortices form between one to three loops at each wing and are occasionally also found at U = 1 m/s in our bats. These vortices reflect thrust generation, which should be important at this intermediate flight speed range since the upstroke generates little weight support. By using the upstroke to generate thrust, the downstroke can be used to mainly generate weight support. However, a likely function of the wing pitch is to control pitch and yaw of the animal. Since the wing pitch occurs with the hand wings high and extended laterally relative to the center of mass, the large moments generated would be efficient to control body rotations^48^. Since we expect the bats to perform minor control maneuvering when flying in the wind tunnel, a maneuvering control function would explain the variation between wingbeats.

The wake of *P. auritus* is found to be more complex than previously recognized in bats, yet also pointing to structures that may be universal to flapping wings, such as the spiraling wing tip vortices. The vortex sheet shed at the trailing edge of the wings roll up to discrete vortices associated with the increasing or decreasing circulation of the accelerating wings during the wingbeat, to yield a spiraling tip vortex. In addition, we propose a novel mechanism to bat flight, where a strong pitch motion at the end of the upstroke at low flight speeds can generate thrust and provide an efficient means to control pitch and yaw. The effectiveness of this mechanism will depend on the pitch rate relative to the forward flight speed and may therefore only be expected at relatively slow flight speeds. The high flapping rate and non-flexed wings should make insects strong candidates for using a similar mechanism of thrust production.

Contrary to previously studied bat species, our results demonstrate a substantial downwash generated behind the body of *P. auritus* at cruising speed. While this can be a direct result of the large ears, our study cannot isolate the aerodynamic effect of the ears from that of the tail membrane. However, the ability to mitigate negative effects of external ears may reduce evolutionary costs and help explain the diversity in bat morphology. Future studies should separate the contribution of ears and tail by, for example, studying aerial hawking vespertilionids (small ears and large tail membrane) and gleaning phyllostomines (large ears and small tail membrane) or bat models. If using models^20^, the negative effects associated with the interaction with the wings found here need to be addressed. Since the negative interaction is highly dynamic, it emphasizes that when using models they should incorporate flapping wings (e.g.^49^). These interaction effects also illuminate the complexity of the adaptive landscape of bat ears.

## Materials and Methods

### Bats

We trained three wild-caught *P. auritus*, to capture a mealworm at a feeder in the wind tunnel at Lund University, Sweden^50^. The bats flew at 1, 2, 3, 4 and 5 m/s and the number of sequences captured for each individual at each speed is shown in Table S1. All animals were weighted after each experimental trial and we obtained morphological information from in-flight photos (Table S2). The study was performed in accordance with approved experimental guidelines. Procedures were approved by the Malmö-Lund animal ethics committee (M 33-13).

### PIV

We measured the flow 10 cm behind the bats using a standard stereo PIV setup (e.g.^51^), with two high speed cameras (LaVision Imager pro HS 4M, 2016 × 2016 pixels) looking obliquely from above at a transverse light sheet (LDY304PIV laser, Litron Lasers Ltd, Rugby, England). Images of particles (1 μm) were captured at f_L_ = 640 Hz when the bats were flying steadily, before approaching the feeder. The imaged area was approximately 30 × 20 cm (width x hight), resulting in ~65 pixels/cm. Cameras were calibrated (type 22 calibration plate), and the images analyzed in Davis 8.2 (LaVision Gmbh, Goettingen, Germany) using a decreasing box size (64 × 64 followed by 32 × 32 boxes with 50% overlap). The resulting vector fields (~4 vectors/cm) were post-processed using a 1× outlier detection and a 2× median-filter to remove and replace erroneous vectors. Empty spaces were filled by interpolation. No smoothing was necessary, since the vector fields were considered to be of high quality.

The images covered more than half the span, but not the full span. We estimated weight support by doubling the integrated vorticity multiplied by the voxel size (within-plane spacing of vectors (dx*dy = 0.0024*0.0024 m) times the flight speed and frame interval [U/f_L_]), the air density (ρ = 1.2 kg/m^3^), and the distance between the voxel and the center of the body (s_Rloc_) for the semi-span. The center of the body was determined manually, as the symmetry plane of the wake structures. The derived weight support was then compared with the weight of the animal and sequences showing weight support ±20% were kept for further analysis (total of 26 sequences).

For illustrations we generated 3D matrices with spacing between vectors in the measurement plane (dx*dy), and U/f_L_ in the out of plane direction. We interpolated the data to acquire a homogenously spaced (dx = dy = dz = 0.0024 m) dataset (interp3 [cubic spline] function in Matlab) and applied a Gaussian smoothing (smooth3 [5, 5, 5] function in Matlab). To identify vortices we calculated the Q-criteria (VortexID function Matlab, written by Martin Kearney-Fischer (https://github.com/ganglere/matlab/blob/master/VortexID.m) and generated isosurface plots of the 3D vortex structures. The Q-criteria estimated in this way is a pseudo-representation, since the volume is based on stacking 2D planes with three component vectors and not a true volume. However, stacking time resolved stereo-images for 3D-representation of the wake generate relatively small errors^52^.

## Additional Information

**How to cite this article**: Johansson, L. C. *et al.* Ear-body lift and a novel thrust generating mechanism revealed by the complex wake of brown long-eared bats (Plecotus auritus). *Sci. Rep.* **6**, 24886; doi: 10.1038/srep24886 (2016).

## Supplementary Material

Supplementary Information

## References

[b1] Simmons N. B. Order Chiroptera. Third edn, 312–529 (Johns Hopkins University Press, 2005).

[b2] Griffin D. R. Listening in the dark. Second edn, (Yale Univ. Press, 1958).

[b3] Johansson L. C., Wolf M. & Hedenström A. A quantitative comparison of bird and bat wakes J. R. Soc. Interface. 7 61–66 (2010). 19324669 10.1098/rsif.2008.0541 PMC2839372

[b4] Muijres F. T., Johansson L. C., Bowlin M. S., Winter Y. & Hedenström A. Comparing aerodynamic efficiency in birds and bats suggests better flight performance in birds. PLOS One 7, e37335, 10.1371/journal.pone.0037335 (2012). 22624018 PMC3356262

[b5] Norberg U. M. & Rayner J. M. V. Ecological morphology and flight in bats (Mammalia; Chiroptera): Wing adaptations, flight performance, foraging strategy and echolocation. Phil. Trans. R. Soc. Lond. B 316, 335–427 (1987).

[b6] Fenton M. B. The structure of aerial-feeding bat faunas as indicated by ears and wing elements. Can. J. Zool. 50, 287–296 (1972).

[b7] Obrist M. K., Fenton M. B., Eger J. L. & Schlegel P. A. What ears do for bats: a comparative study of pinna sound pressure transformation in chiroptera. J. Exp. Biol. 180, 119–152 (1993). 8371084 10.1242/jeb.180.1.119

[b8] Gardiner J. D., Codd J. R. & Nudds R. L. An association between ear and tail morphologies of bats and their foraging style. Can. J. Zool. 89, 90–99, 10.1139/Z10-096 (2011).

[b9] Schnitzler H.-U. & Kalko E. K. V. Echolocation by insect-eating bats. BioScience 51, 557–569 (2001).

[b10] Hubel T. Y., Riskin D. K., Swartz S. M. & Breuer K. S. Wake structure and wing kinematics: the flight of the lesser dog-faced fruit bat, Cynopterus brachyotis. J. Exp. Biol. 213, 3427–3440 (2010). 20889823 10.1242/jeb.043257

[b11] Hubel T. Y., Hristov N. I., Swartz S. M. & Breuer K. S. Changes in kinematics and aerodynamics over a range of speeds in *Tadarida brasiliensis*, the Brazilian free-tailed bat. J. R. Soc. Interface. 9, 1120–1130 (2012). 22258554 10.1098/rsif.2011.0838 PMC3350744

[b12] Hedenström A. *et al.* Bat flight generates complex aerodynamic tracks. Science 316, 894–897 (2007). 17495171 10.1126/science.1142281

[b13] Johansson L. C. *et al.* The near and far wake of Pallas’ long tongued bat (*Glossophaga soricina*). J. Exp. Biol. 211, 2909–2918 (2008). 18775928 10.1242/jeb.018192

[b14] Johansson L. C. & Hedenström A. The vortex wake of blackcaps (*Sylvia atricapilla* L.) measured using high-speed digital particle image velocimetry (DPIV). J. Exp. Biol. 212 3365–3376 (2009). 19801441 10.1242/jeb.034454

[b15] Henningsson P., Muijres F. T. & Hedenström A. Time-resolved vortex wake of a common swift flying over a range of flight speeds. J. R. Soc. Interface, rsif20100533, 10.1098/rsif.2010.0533 (2010). PMC3104350 21131333

[b16] Muijres F. T., Bowlin M. S., Johansson L. C. & Hedenström A. Vortex wake, downwash distribution, aerodynamic performance and wingbeat kinematics in slow-flying pied flycatchers. J. R. Soc. Interface 9, 292–303 (2012). 21676971 10.1098/rsif.2011.0238 PMC3243385

[b17] von Busse R., Waldman R. M., Swartz S. M., Voigt C. C. & Breuer K. S. The aerodynamic cost of flight in the short-tailed fruit bat (*Carollia perspicillata*): comparing theory with measurement. J. R. Soc. Interface 11, 20140147, 10.1098/rsif.2014.0147 (2014). PMC4006254 24718450

[b18] Hedenström A. & Johansson L. C. Bat flight: aerodynamics, kinematics and flight morphology. J. Exp. Biol. 218, 653–663, 10.1242/jeb.031203 (2015). 25740899

[b19] Gardiner J. D., Dimitriadis G., Sellers W. I. & Codd J. R. The aerodynamics of big ears in the brown long-eared bat Plecotus auritus. Acta Chiropterologica 10, 313–321, 10.3161/150811008X414881 (2008).

[b20] Vanderelst D., Peremans H., Razak N. A., Verstraelen E. & Dimitriadis G. The Aerodynamic Cost of Head Morphology in Bats: Maybe Not as Bad as It Seems. Plos ONE 10, e0118545, 10.1371/journal.pone.0118545 (2015). 25739038 PMC4349651

[b21] Von Busse R., Hedenström A., Winter Y. & Johansson L. C. Kinematics and wing shape across flight speed in the bat, Leptonycteris yerbabuenae. Biology Open 1, 1226–1238, 10.1242/bio.20122964 (2012). 23259057 PMC3522884

[b22] Hedenström A. & Johansson L. C. Bat flight. Curr. Biol. 25, R399–R402, 10.1016/j.cub.2015.04.002 (2015). 25989074

[b23] Swartz S. M. & Konow N. Advances in the study of bat flight: the wing and the wind. Canadian Journal of Zoology 93, 977–990, 10.1139/cjz-2015-0117 (2015).

[b24] Muijres F. T., Johansson L. C., Winter Y. & Hedenström A. Leading edge vortices in lesser long-nosed bats occurring at slow but not fast flight speeds. Bioinsp. Biomim. 9, 025006 (2014). 24855067 10.1088/1748-3182/9/2/025006

[b25] Wolf M., Johansson L. C., von Busse R., Winter Y. & Hedenström A. Kinematics of flight and the relationship to the vortex wake of a Pallas’ long tongued bat (*Glossophaga soricina*). J. Exp. Biol. 213, 2142–2153 (2010). 20511529 10.1242/jeb.029777

[b26] Norberg U. M. Aerodynamics, kinematics, and energetics of horizontal flapping flight in the Long eared bat *Plecotus auritus*. J. exp. Biol. 65, 179–212 (1976). 993701 10.1242/jeb.65.1.179

[b27] Dickinson M. H., Lehmann F.-O. & Sane S. P. Wing rotation and the aerodynamic basis of insect flight. Science 284, 1954–1960 (1999). 10373107 10.1126/science.284.5422.1954

[b28] Weis-Fogh T. Quick estimates of flight fitness in hovering animals, including novel mechanisms for lift production. J. Exp. Biol. 59, 169–230 (1973).

[b29] Muijres F. T. *et al.* Leading-edge vortex improves lift in slow-flying bats. Science 319, 1250–1253, 10.1126/science.1153019 (2008). 18309085

[b30] Muijres F. T., Johansson L. C., Winter Y. & Hedenström A. Comparative aerodynamic performance of flapping flight in two bat species using time-resolved wake visualization. J. R. Soc. Interface. 8, 1418–1428 (2011). 21367776 10.1098/rsif.2011.0015 PMC3163419

[b31] Håkansson J., Hedenström A., Winter Y. & Johansson L. C. The wake of hovering flight in bats. J. R. Soc. Interface 12, 20150357, 10.1098/rsif.2015.0357 (2015). PMC4535406 26179990

[b32] Gardiner J. D., Dimitriadis G., Codd J. R. & Nudds R. L. A Potential Role for Bat Tail Membranes in Flight Control. Plos ONE 6, e18214, 10.1371/journal.pone.0018214 (2011). 21479137 PMC3068189

[b33] Altaf A., Omar A. A., Asrar W. & Ludin Jamaluddin H. B. Study of the Reverse Delta Wing. J. Aircraft 48, 277–286, 10.2514/1.C031101 (2011).

[b34] Anderson J. D. Fundamentals of aerodynamics. 4^th^ edn, (McGraw-Hill, 2007).

[b35] Henningsson P. *et al.* The complex aerodynamic footprint of desert locusts revealed by large-volume tomographic particle image velocimetry. J. R. Soc. Interface 12, 20150119, 10.1098/rsif.2015.0119 (2015). PMC4528577 26040598

[b36] Harbig R. R., Sheridan J. & Thompson M. C. Reynolds number and aspect ratio effects on the leading-edge vortex for rotating insect wing planforms. J. Fluid Mech. 717, 166–192 (2013).

[b37] Freymuth P., Finaish F. & Bank W. Further visualization of combined wing tip and starting vortex systems. AIAA Journal 25, 1153–1159 (1987).

[b38] Visbal M. In 49th AIAA Aerospace Sciences Meeting including the New Horizons Forum and Aerospace Exposition Aerospace Sciences Meetings (American Institute of Aeronautics and Astronautics, 2011).

[b39] Bross M., Ozen C. A. & Rockwell D. Flow structure on a rotating wing: Effect of steady incident flow. Physics of Fluids 25, 081901, 10.1063/1.4816632 (2013).

[b40] Johansson L. C., Engel S., Kelber A., Klein Heerenbrink M. & Hedenstrom A. Multiple leading edge vortices of unexpected strength in freely flying hawkmoth. Sci. Rep. 3, 3264, 10.1038/srep03264 (2013). 24253180 PMC3834544

[b41] Aldridge H. D. J. N. Kinematics and aerodynamics of the Greater horseshoe bat, *Rhinolophus ferrumequinum*, in horizontal flight at various flight speeds. J. Exp. Biol. 126, 479–497 (1986). 3806000 10.1242/jeb.126.1.479

[b42] Weihs D. The mechanism of rapid starting of slender fish. Biorheology 10, 343–350 (1973). 4772008 10.3233/bir-1973-10308

[b43] Witt W. C., Wen L. & Lauder G. V. Hydrodynamics of C-Start Escape Responses of Fish as Studied with Simple Physical Models. Integrative and Comparative Biology, 10.1093/icb/icv016 (2015). 25920507

[b44] Borazjani I. Simulations of Unsteady Aquatic Locomotion: From Unsteadiness in Straight-Line Swimming to Fast-Starts. Integ. and Comp. Biol., icv015, 10.1093/icb/icv015 (2015). 25888943

[b45] Ol M. In 39th AIAA Fluid Dynamics Conference Fluid Dynamics and Co-located Conferences (American Institute of Aeronautics and Astronautics, 2009).

[b46] Buchner A. J. & Soria J. Measurements of the flow due to a rapidly pitching plate using time resolved high resolution PIV. Aerospace Science and Technology 44, 4–17, 10.1016/j.ast.2014.04.007 (2015).

[b47] Johansson L. C. & Norberg R. Å. Delta-wing function of webbed feet gives hydrodynamic lift for swimming propulsion in birds. Nature 424, 65–68 (2003). 12840759 10.1038/nature01695

[b48] Taylor G. K. & Thomas A. L. R. Animal flight dynamics II. Longitudal stability in flapping flight. J. theor. Biol. 214, 351–370 (2002). 11846595 10.1006/jtbi.2001.2470

[b49] Bahlman J. W., Swartz S. M. & Breuer K. S. How wing kinematics affect power requirements and aerodynamic force production in a robotic bat wing. Bioinspiration & Biomimetics 9, 025008, 10.1088/1748-3182/9/2/025008 (2014). 24851830

[b50] Pennycuick C. J., Alerstam T. & Hedenström A. A new low-turbulence wind tunnel for bird flight experiments at Lund University, Sweden. J. Exp. Biol. 200, 1441–1449 (1997). 9319339 10.1242/jeb.200.10.1441

[b51] Johansson L. C. *et al.* Elytra boost lift, but reduce aerodynamic efficiency in flying beetles. J. R. Soc. Interface 9, 2745–2748 (2012). 22593097 10.1098/rsif.2012.0053 PMC3427496

[b52] Bomphrey R. J., Henningsson P., Michaelis D. & Hollis D. Tomographic particle image velocimetry of desert locust wakes: instantaneous volumes combine to reveal hidden vortex elements and rapid wake deformation. J. R. Soc. Interface 9, 3378–3386, 10.1098/rsif.2012.0418 (2012). 22977102 PMC3481570

