# Supplementary material for: Ear-body lift and a novel thrust generating mechanism revealed by the complex wake of brown long-eared bats (Plecotus auritus)
Source: Sci Rep. 2016 Apr 27;6:24886. doi: 10.1038/srep24886 (PMC4846812; doi:10.1038/srep24886)
Supplement: Supplementary Information [file srep24886-s1.zip › Johansson et al_SI.pdf]

Ear-body lift and a novel thrust generating mechanism revealed by the complex wake of brown long-eared bats (Plecotus auritus)

L. Christoffer Johansson, Jonas Håkansson, Lasse Jakobsen and Anders Hedenström

Corresponding author: Christoffer Johansson, Dept. Biology, Lund University, Ecology building, SE-223 62 Lund, Sweden. Phone +46 46 222 4955 Email: Christoffer.Johansson@biol.lu.se

Table S1.

Number of sequences analyzed at each flight speed for each bat.

Bat\Speed	1 m/s	2 m/s	3 m/s	4 m/s	5 m/s	Sum
RS	-	3	1	-	1	5
T	1	2	3	3	3	12
LS	1	2	1	3	2	9
Sum	2	7	5	6	6	26

Table S2.

Morpholgical measurements of the three *P. auritus* used.

Bat	M (g)	b (m)	S (m^2)	c (m)	AR	Q (N/m^2)
RS	8.7	0.302	0.015496	0.051	5.9	5.5
T	9.2	0.295	0.014626	0.050	6.0	6.2
LS	9.0	0.278	0.013064	0.047	5.9	6.8

Span (b) refers to the tip to tip span during mid downstroke when flying at 4 m/s. Wing area (S) is measured including the projected body area between the wings. Mean chord (c) is S/b and aspect ratio (AR) b^2/ S. Wing loading (Q) is Mg/S.

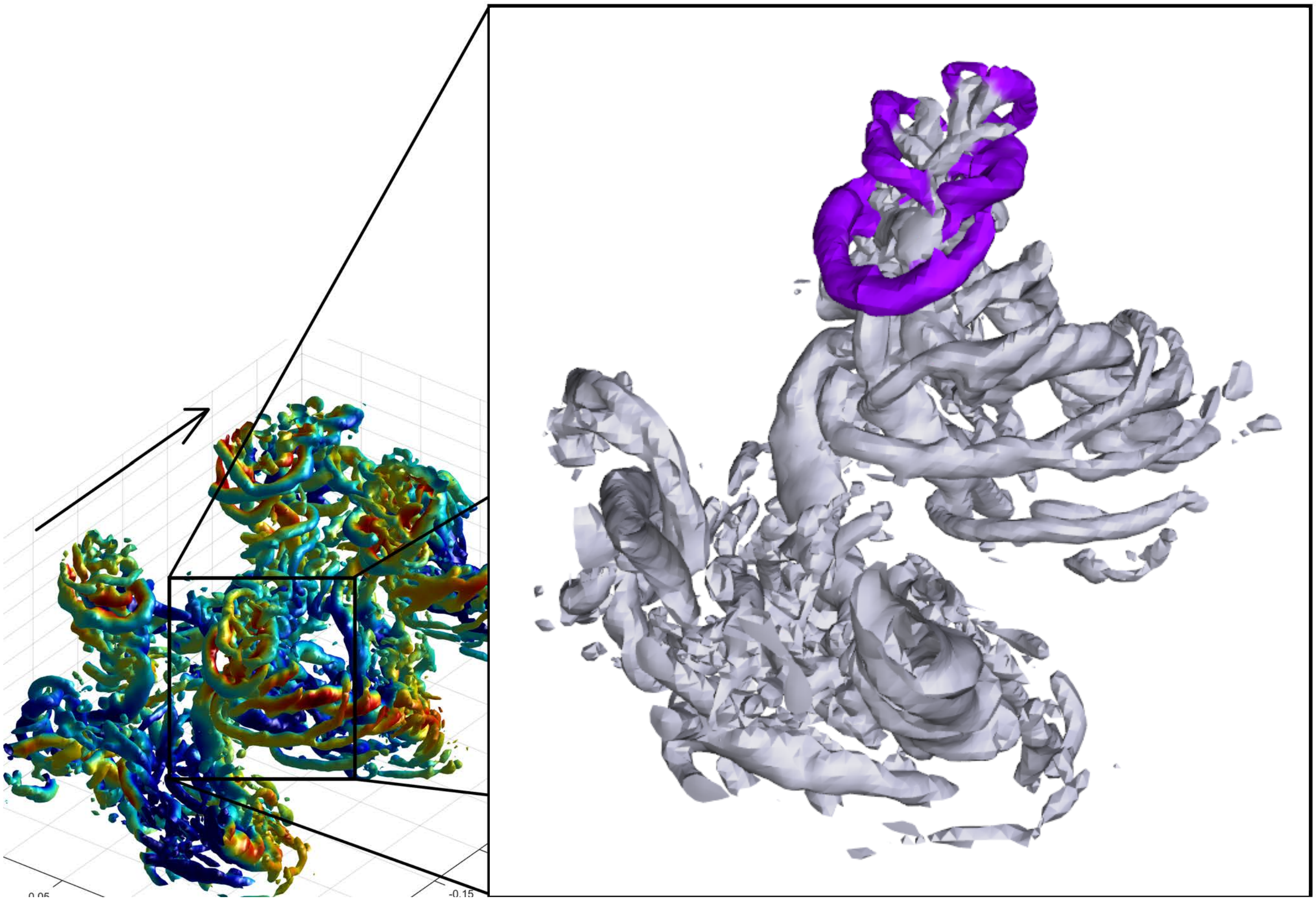

Figure S1. Iso-surface plot of Q-criteria (2500) showing the vortices generated at the transition between upstroke and downstroke at 2 m/s, viewed obliquely from above and behind. Rotatable zoomed in view with vortex structures showing upwards and backwards induced flow, formed as the wing performs a pronating, pitch down, motion at the transition are colored purple. Flight direction is indicated by arrow.

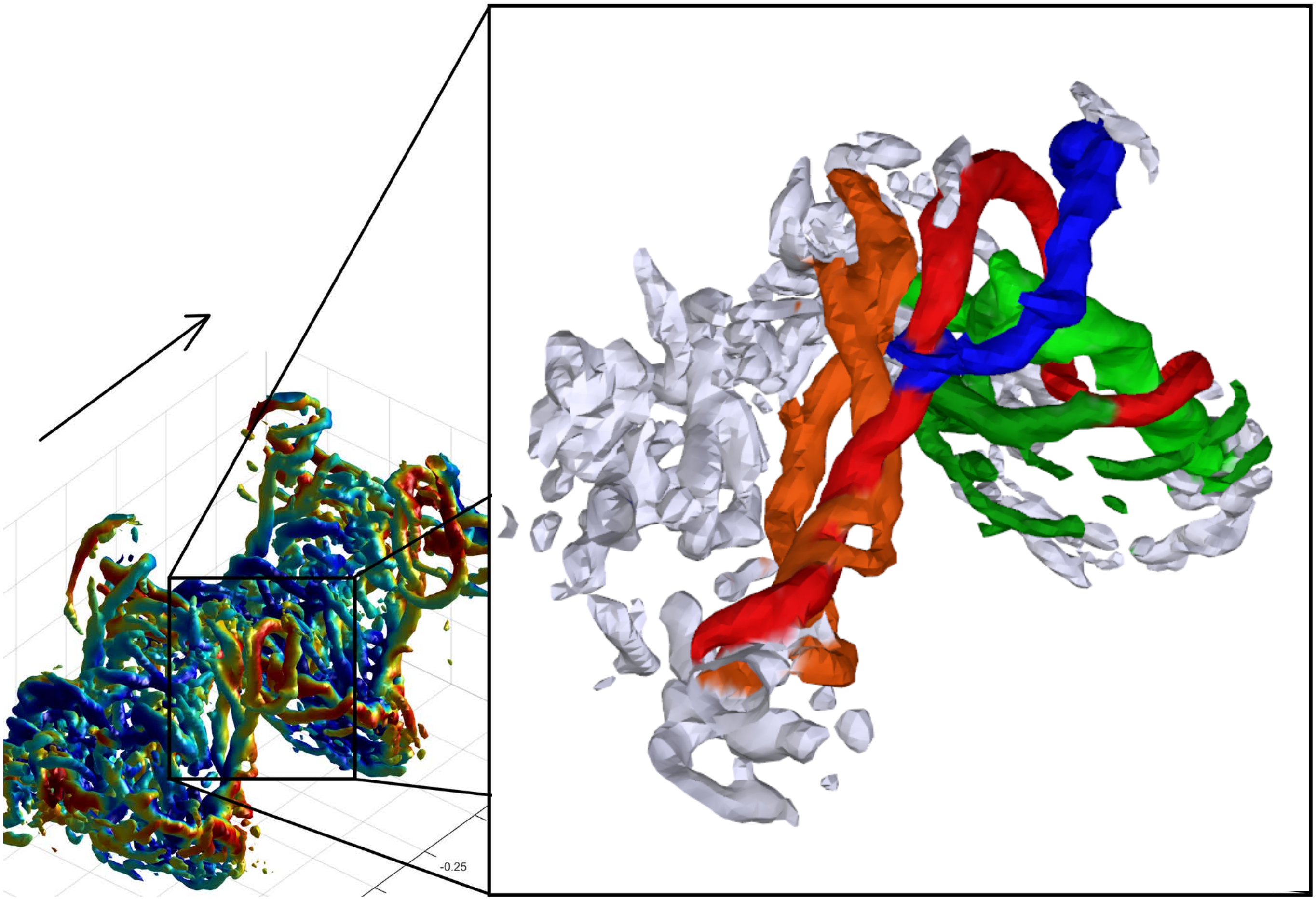

Figure S2. Iso-surface plot of Q-criteria (2500) showing the details of vortices generated at the transition between upstroke and downstroke at 3 m/s, viewed obliquely from above and behind. Rotatable zoomed in view with the tip vortex (red), reduces in strength during the upstroke resulting in shedding of stop vortices (orange). Towards the end of the upstroke the wing tip sheds a tip vortex (blue) of opposite sense of rotation to the normal tip vortex. At the beginning of the next downstroke, a start vortex is shed (light green). The circulation builds up during the downstroke as indicated by additional start sense vortices (dark green) being shed. The resulting tip vortex constitutes several vortices spiraling around each other. Flight direction indicated by arrow.

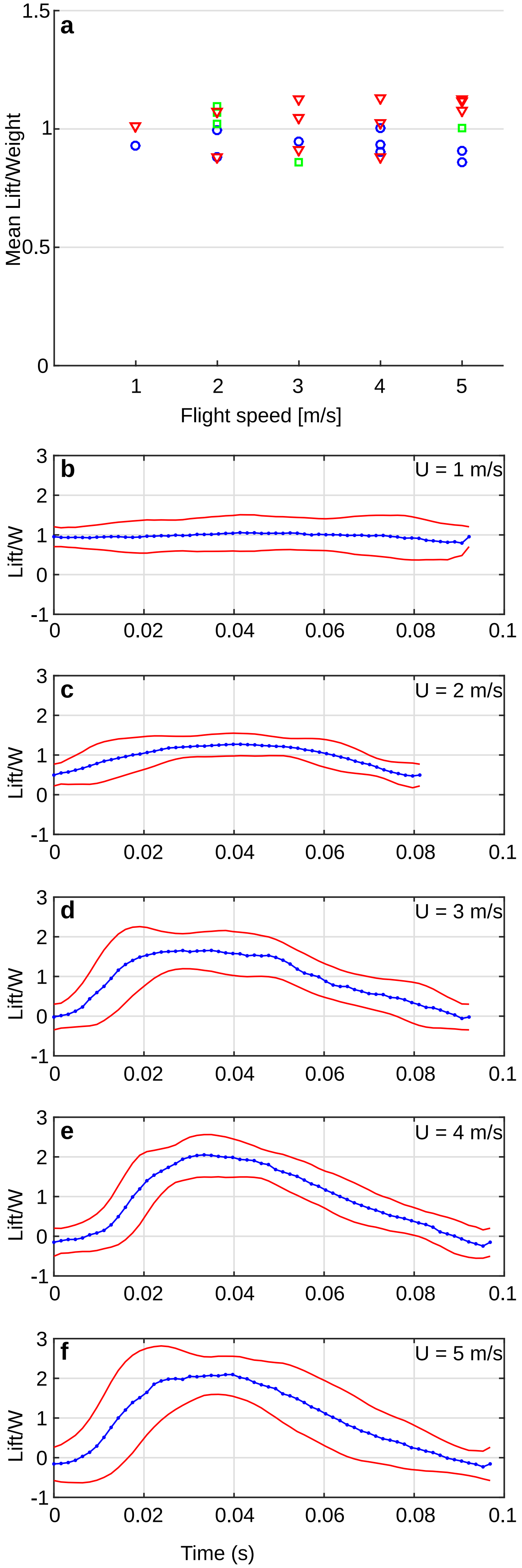

Figure S3. Mean vertical force throughout a sequence across flight speeds for the sequences used in this study. The colors/symbols represent the different individuals (a). The vertical force measured in the wake varies throughout the wingbeat (b-f) with negative vertical force generated during the upstroke at 3-5 m/s. The blue lines represents the normalized vertical force for all the wingbeats at a particular speed and the red lines the +- 2* SE, estimated using a repeated measures setup to account for several measurements for each individual and sequence (Matlab, anovan function)

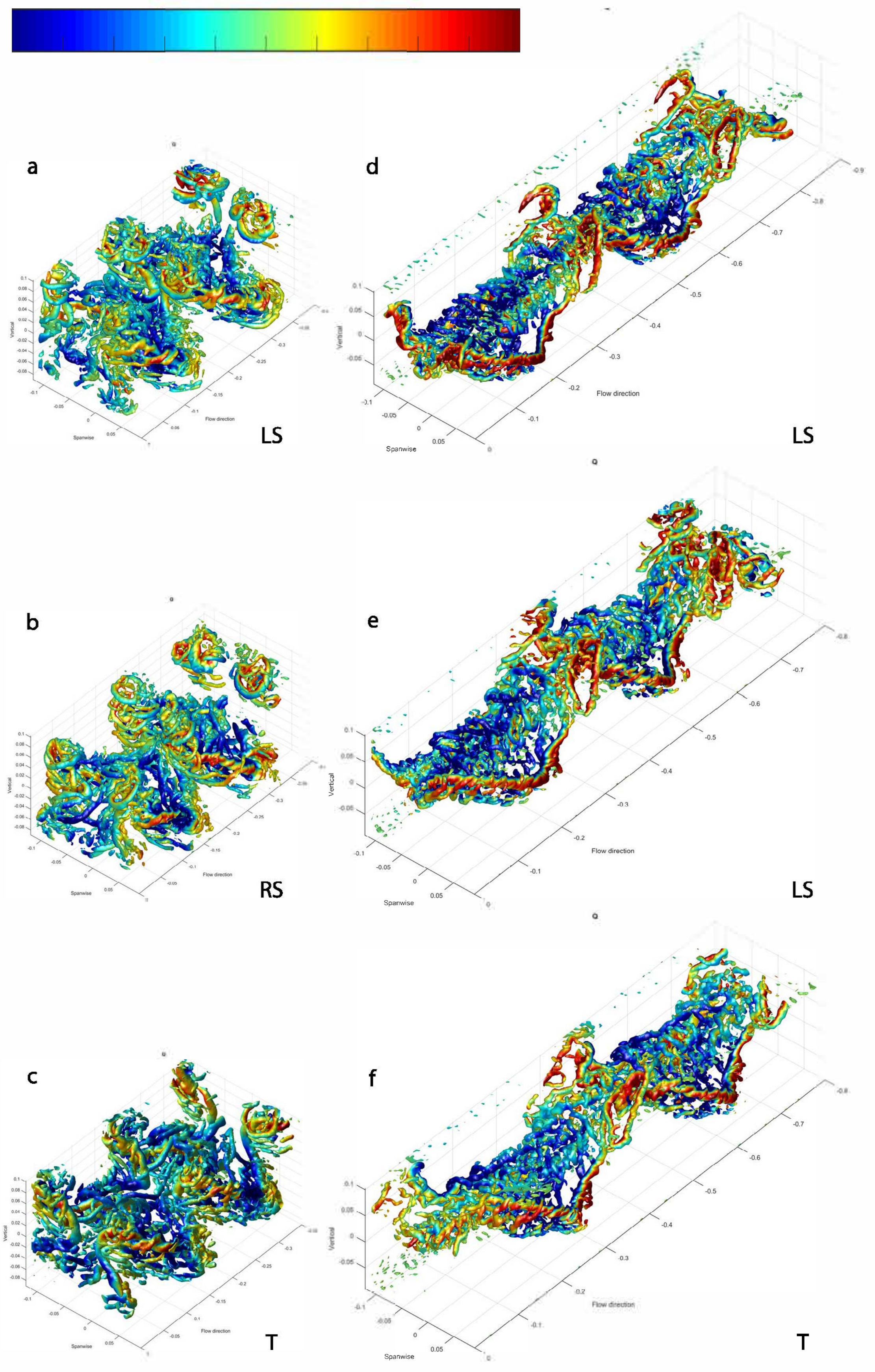

Figure S4. Sample sequences of wakes visualized by Q-criteria indicating the variation in the data. Vortices are colored by vertical speed according to the colorbar +-1 = 3*std of the vertical flow in the measured volume. **a-c** showing 2 m/s flight speed and **d-f** showing 4 m/s. In **a** max velocity for scaling is 1.32 m/s, in **b** 1.27 m/s, in **c** 1.45 m/s, in **d** 0.96 m/s, in **e** 0.91 m/s and in **f** 0.93 m/s. LS, RS and T refers to the different individuals (Table S1). Flight direction is to the right and into the image.
