# Supplementary material for: Ear-body lift and a novel thrust generating mechanism revealed by the complex wake of brown long-eared bats (Plecotus auritus)
Source: Sci Rep. 2016 Apr 27;6:24886. doi: 10.1038/srep24886 (PMC4846812; doi:10.1038/srep24886)
Supplement: Supplementary Information [file srep24886-s1.zip › Johansson et al_Fig S5.pdf]

Ear-body lift and a novel thrust generating mechanism revealed by the complex wake of brown long-eared bats (Plecotus auritus)

L. Christoffer Johansson, Jonas Håkansson, Lasse Jakobsen and Anders Hedenström

Corresponding author: Christoffer Johansson, Dept. Biology, Lund University, Ecology building, SE-223 62 Lund, Sweden. Phone +46 46 222 4955 Email: Christoffer.Johansson@biol.lu.se

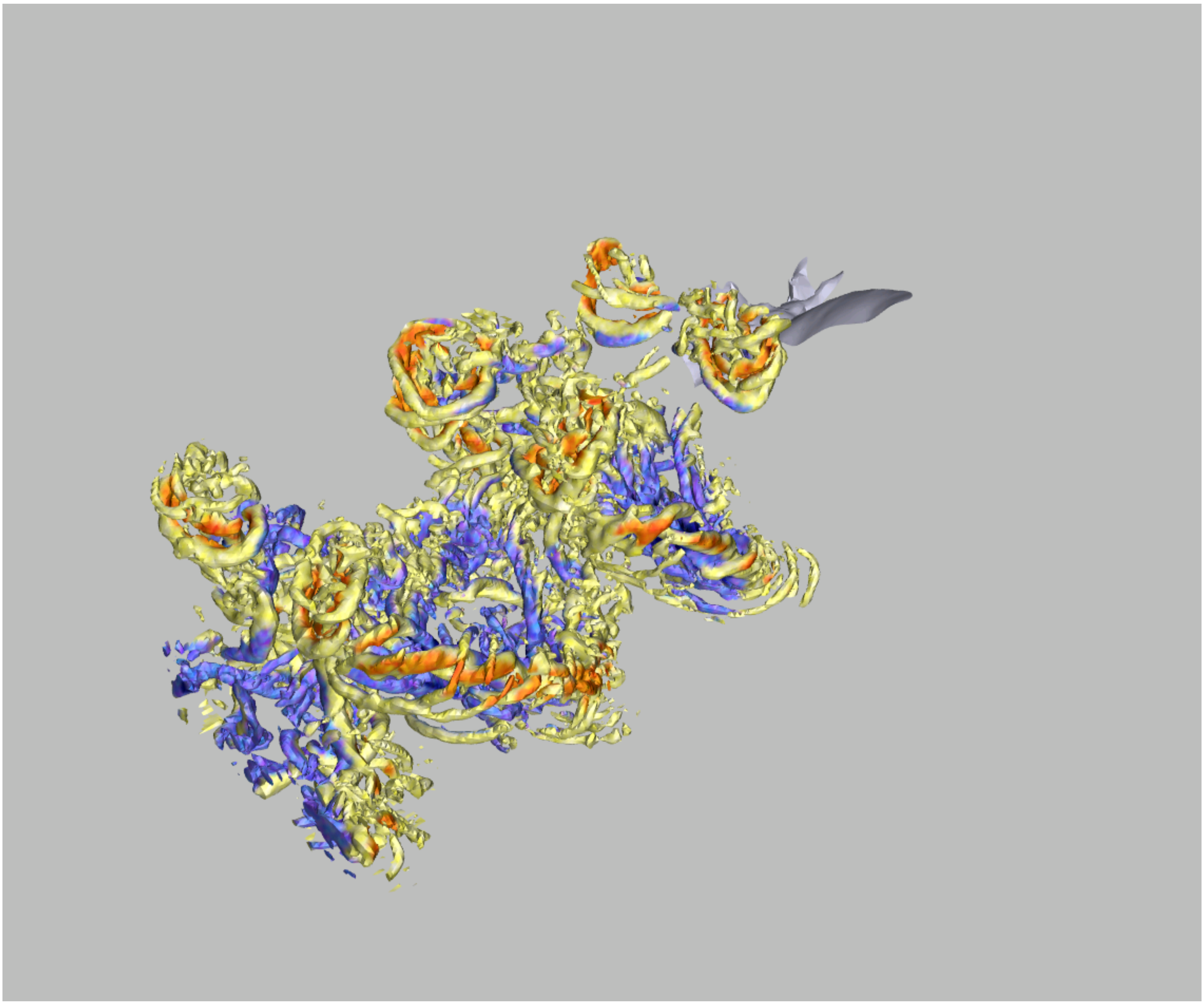

Figure S5. Rotatable wakes visualized by isosurfaces (2500) of Q-criteria at 2.5 m/s showing approximately two wingbeats. Vortices are colored by vertical speed normalized by 2.5 m/s, red indicating upwash and blue downwash. The bat indicates flight direction.
